# Draft Genome of *Busseola fusca*, the Maize Stalk Borer, a Major Crop Pest in Sub-Saharan Africa

**DOI:** 10.1093/gbe/evz166

**Published:** 2019-07-31

**Authors:** Kayla M Hardwick, Awino Maureiq Edith Ojwang', Francesca Stomeo, Solomon Maina, Gladys Bichang’a, Paul-André Calatayud, Jonathan Filée, Appolinaire Djikeng, Caitlin Miller, Leah Cepko, Alistair C Darby, Bruno Le Ru, Sarah Schaack

**Affiliations:** 1 Department of Biology, Reed College, Portland, Oregon; 2 Phylos Bioscience, Portland, Oregon; 3 Biosciences eastern and central Africa - International Livestock Research Institute (BecA-ILRI) Hub, Nairobi, Kenya; 4 Department of Biochemistry, University of Nairobi, Kenya; 5 International Centre of Insect Physiology and Ecology (icipe), Nairobi, Kenya; 6 Evolution, Génomes, Comportement, Ecologie, CNRS, IRD, Université Paris-Sud, Université Paris-Saclay, Gif-sur-Yvette, France; 7 Centre for Genomic Research, Institute of Integrative Biology, University of Liverpool, Liverpool, United Kingdom; Biomathematics Graduate Program, North Carolina State University, Raleigh, NC; European Molecular Biology Laboratory (EMBL), Heidelberg, Germany; Agriculture Victoria Research, Horsham, Victoria, Australia; Centre for Tropical Livestock Genetics and Health, The University of Edinburgh, Scotland, United Kingdom; Department of Biology, Cornell University, Ithaca, NY

**Keywords:** agricultural pest, Lepidoptera, insect genomics

## Abstract

The maize stalk borer, *Busseola fusca*, is an important Lepidopteran pest of cereal crops in Central, East, and Southern Africa. Crop losses due to *B. fusca* feeding activity vary by region, but can result in total crop loss in areas with high levels of infestation. Genomic resources provide critical insight into the biology of pest species and can allow for the development of effective management tools and strategies to mitigate their impact on agriculture. To this end, we sequenced, assembled, and annotated the genome of *B. fusca*. The total assembled genome size was 492.9 Mb with 19,417 annotated protein-coding genes. Using a comparative approach, we identified a putative expansion in the Chorion gene family, which is involved in the formation of the egg shell structure. Our analysis revealed high repeat content within the *B. fusca* genome, with LTR sequences comprising the majority of the repetitive sequence. We hope genomic resources will provide a foundation for future work aimed at developing an integrated pest management strategy to reduce *B. fusca’s* impact on food security.

## Introduction

The maize stalk borer, *Busseola fusca* (Fuller) (Lepidoptera, Noctuidae; supplementary fig. S1, Supplementary Material online), is an important pest of cereal crops in Central, East, and Southern Africa. Due to its abundance and distribution, it represents the major constraint to the production of maize and sorghum in many regions of sub-Saharan Africa (Kfir et al. 2002). Its impact on the food security and economic well-being of people in this region has made it the subject of intense study (reviewed in Calatayud et al. 2006). While whole genome sequencing projects can provide basic information about genomic content, they can also provide insight into important traits related to insecticide resistance, adaptation to plant defense mechanisms, immunity, and chemoreception which can, in turn, inform or enhance management strategies for pest species (e.g., *Plutella xylostella*; You et al. 2013).

Crop losses due to *B. fusca* feeding activity vary by region, but can result in a total loss in areas with major infestations (Van den Berg et al. 1991; Calatayud et al. 2014). Females typically deposit eggs between the stem and leaf sheet of the host plant. Larvae hatch, feed on young leaves, and penetrate the plant stem during the third instar, where they remain until pupation. Feeding during the larval stage, which is also when the animals are vulnerable to parasitoid wasps, damages the host plant and reduces yield or kills the plant. After pupation, adult moths use chemosensory cues and receptors to attract and find mates, food, and suitable places to lay eggs. Useful pest management strategies, therefore, include introducing substances that can interrupt sending chemical cues or their reception. 

Another cost-effective strategy for pest management, championed because it involves fewer chemicals, is biological control. In the 1990s, a biocontrol program was launched in Kenya to try and manage *B. fusca* populations using the wasp, *Cotesia sesamiae* (Cameron; Hymenoptera: Braconidae), an indigenous larval parasitoid of *B. fusca*. Parasitism by *C. sesamiae* can vary (ranging from <5% to 75%; Kfir 1995; Sallam et al. 1999; Jiang et al. 2006; Songa et al. 2007) in part due to differences among strains (Mochiah et al. 2002; Gitau et al. 2010; Branca et al. 2011). *Busseola fusca* are resistant to infection by *C. sesamiae* from Mombasa (coastal Kenya), but vulnerable to *C. sesamiae* from Kitale (a site in inland Kenya; Ngi-Song et al. 1995). The genetic basis of differences in host immunity and susceptibility to infection among species and strains could provide helpful insight for future biocontrol programs.

Despite the major economic importance of *B. fusca*, little is known about the genetics and genomics of this species other than phylogeographic studies (Sezonlin et al. 2006; Dupas et al. 2014) and the report of dominant inheritance of field-evolved resistance to *Bt* maize (Campagne et al. 2013). Here, we sequenced, assembled, and annotated the genome of *B. fusca* to characterize its content and identify important candidate genes or gene families for ongoing management efforts and future research aimed at curbing the effects of this devastating crop pest.

## Materials and Methods

Animal rearing, tissue collection, library preparation, and sequencing were all performed at the International Centre of Insect Physiology and Ecology (*icipe*) and Biosciences eastern and central Africa (BecA)-Hub genomics facility at the International Livestock Research Institute (ILRI) in Nairobi, Kenya (see supplementary Methods M1 for detailed methods, Supplementary Material online). 

Briefly, specimens were obtained from a colony of *B. fusca* initiated from larvae collected in Western Province of Kenya in 2008. Extractions of total genomic DNA were performed using 6 legs from an adult male. Sequencing two DNA libraries using the Illumina MiSeq platform (see supplementary Methods M2 for details on library preparation, sequencing, and assembly, Supplementary Material online) resulted in 112,971,972 raw reads (∼70× coverage) for the genome (supplementary table S1, Supplementary Material online). Scaffolding and decontamination steps are outlined in the supplementary Methods M2, Supplementary Material online. As an indication of the quality of our assembly, the number of contigs/scaffolds, total assembly size, maximum scaffold size, N50 value, median contig length, and GC content (using QUAST, v. 4.5; Gurevich et al. 2013) are summarized in table 1. Raw reads are available in the short read archive at National Center for Biotechnology Information (PRJNA553865).

**Table 1:** Genome Assembly Statistics for *B. fusca* (v. 1.0; accession VKGM00000000)

	Genome (Contigs)	Genome (Scaffolds)
Number of sequences	250,754	201,397
Total size (bp)	493,968,139	492,902,885
Largest sequence (bp)	36,840	94,607
Sequence N50 (bp)	2,721	3,310
Median sequence length (bp)	1,492	1,604
% GC	39.5	39.5
% BUSCO genes—Eukaryota	81.6	92.0
% BUSCO genes—Arthropoda	83.5	91.1
% BUSCO genes—Insecta	79.6	89.0
Complex repeats (bp)	—	249,564,483
Simple repeats (bp)	—	2,847,630

We analyzed our draft genome using a number of programs (see supplementary Methods M3 for details, Supplementary Material online). First, we ran BUSCO (v. 3; Waterhouse et al. 2018) to assess the completeness of the assembly (table 1) and to compare it to a subset of other Lepidoptera species that have been sequenced (supplementary table S2, Supplementary Material online). We used MAKER (v. 2.3; Cantarel et al. 2008) to annotate the genome using an iterative approach (3 passes) to train gene prediction algorithms. We quantified the distribution of genes within different gene ontology (GO) categories using WEGO (version 2.0; Ye et al. 2018; supplementary fig. S2, Supplementary Material online). To compare the *B. fusca* genome with those of other Lepidopterans, we used OrthoMCL and Venny to compare our set of MAKER-annotated proteins with the proteomes of *Manduca sexta*, *Bombyx mori*, and *P.xylostella* (supplementary fig. S3, Supplementary Material online). We compared gene families among Lepidopteran genomes using OrthMCL (version 2.0.9; Li et al. 2003) to identify putative expansions and look for similarities among species. We generated a comprehensive library of repetitive elements (Class I and II) to characterize the repeat content in the genome (supplementary data file S5, Supplementary Material online) and to mask the genome for gene annotation.

## Results

We were able to identify 92% of conserved orthologous genes from the Eukaryota database (see table 1; supplementary tables S2 and Results R1, Supplementary Material online) in our draft genome for *B. fusca* (v. 1.0; accession VKGM00000000; the version described in this paper is VKGM01000000). Our genome annotation revealed 19,417 protein-coding genes (median size = 2,336 bp [71–50,009 bp] and mean number of exons = 3.7; see supplementary data files S1 and S2, Supplementary Material online for genome annotations and MAKER-identified protein sequences), which falls within the range of values for previously sequenced Lepidopterans (supplementary table S3, Supplementary Material online).

Using OrthoMCL, we identified 4,586 gene clusters shared among all four species (*B. fusca, B. mori*, *M. sexta*, and *P. xylostella*). Of the gene clusters shared among Lepidopteran species, we identified 4 in which *B. fusca* appears to have undergone gene family size expansion (supplementary data file S3, Supplementary Material online). Notably, one cluster contained 29 *B. fusca* proteins with significant homology to *B. mori* Chorion class CA sequences. Supplementary fig. S3, Supplementary Material online illustrates the protein sequences unique to and shared between *B. fusca* and *B. mori*, *M. sexta*, and *P. xylostella*.

We identified 1,120 *B. fusca*-specific genes in 285 clusters (supplementary data file S4, Supplementary Material online), including a number of clusters with possible function related to silk production (e.g., 5 genes with significant homology to Chymotrypsin inhibitor proteins and 5 with homology to Serine protease proteins). We identified unique clusters with potential immune function (e.g., 31 genes in 3 clusters with homology to Hemolymph lipopolysaccharide-binding protein). We also identified *B. fusca*-specific genes with potential roles in pheromone biosynthesis (i.e., three clusters with 25 genes exhibiting significant similarity to various fatty acid synthase genes; supplementary data file S4, Supplementary Material online).

We found that 245.5 Mb (of 49.81%) of the *B. fusca* genome is repetitive, which is higher than most other sequenced Lepidopterans (supplementary table S3, Supplementary Material online). The repeat library composed of 2,102 Class I retrotransposons and 901 Class II DNA transposons (supplementary table S4 and data file S5, Supplementary Material online).

## Discussion

Public health, environmental security, and economic development in sub-Saharan Africa depend, in large part, on food production. The successful management of agricultural pests that target major crop species in the region, thus, plays an important role in trying to slow the widening gap between food production and demand in this region, in particular, and more globally (World Bank 2008). As advances in biotechnology continue, it is an ethical imperative that we devote energy and attention to build and support the development of genomic resources in order to address these challenges. Here, we sequenced, assembled, and annotated the genome of *B. fusca*. Through this work, we have gained insight into the genome biology of this species and have identified a number of genes of interest for pest management applications.

The genome size (table 1) of *B. fusca* is very close to the previously estimated size (1C = 481.5 Mb; Calatayud et al. 2016) and within the range of previously sequenced Lepidopteran genomes (227 Mb in *Papilio polytes* to 824 Mb in *Chilo suppressalis*; supplementary table S3, Supplementary Material online). The number of genes (19,417) is also comparable to that of other sequenced genomes and there are a number of similarities between *B. fusca* and other sequenced Lepidopterans (*B. mori*, *M. sexta*, and *P. xylostella)* that reflect the phylogenetic distances among these taxa (supplementary fig. S3, Supplementary Material online). While the total number of BUSCO genes recovered was relatively high (table 1), the percent of complete BUSCO genes recovered reflects the relatively large number of contigs in our draft genome assembly (supplementary table S2, Supplementary Material online). This could be due, in part, to the high repeat content in *B. fusca* (table 1; supplementary tables S3 and S4, Supplementary Material online), but is also likely to be explained the level of coverage (∼70×) and lack of multiple insert size libraries (e.g., BAC clones and fosmids), such as those used in the other Lepidopteran projects summarized in supplementary table S2, Supplementary Material online. Combining our data set with other short- or long-read data sets, as they become available, will likely increase the N50 and reduce the number of contigs, thereby improving the assembly and completeness of the *B. fusca* genome for future study.

We found evidence for a gene family expansion in *B. fusca* in genes with significant homology to Chorion class CA protein (supplementary data file S3, Supplementary Material online). Such an expansion may be related to the formation of complex egg shell structures, as well as local adaptation to environment-specific selection pressures or to predation (Lecanidou et al. 1986; Regier et al. 1995). We also discovered a number of *B. fusca*-specific gene families (supplementary data file S4, Supplementary Material online). Three of the *B. fusca*-specific gene clusters contained sequences annotated as fatty acid synthase genes (supplementary data file S4, Supplementary Material online). Fatty acids are an important component of insect pheromones, and the ovipositors of moths have been shown to be involved in both chemosensory perception and pheromone biosynthesis (Xia et al. 2015). Because *B. fusca* have been shown to utilize chemical cues when searching for oviposition sites (Juma et al. 2016), future work should look at the importance of these unique sequences in intraspecific communication and selection of suitable egg-laying sites in *B. fusca* as a part of a comprehensive pest management strategy.

Other potential genes of interest that we annotated were those with functions linked to silk metabolism (e.g., trypsin and serine protease genes; supplementary data file S5, Supplementary Material online). Neonates produce silk in order to aid in dispersal from one host plant to another via “ballooning” (Kafatos et al. 1967; Van Rensburg et al. 1987). Again, future work investigating the role of these gene families in contributing to *B. fusca* dispersal could provide an important pest management strategy that has not previously received attention.

Our protein clustering analysis revealed genes with significant homology to Hemolymph lipopolysaccharide-binding protein (supplementary data file S5, Supplementary Material online), and it is known that hemocytes mediate defense mechanisms such as phagocytosis and encapsulation (Salt 1973) in response to parasitoid wasp infection in insects such as *D. melanogaster*. *Cotesia sesamiae* females deposit their eggs within *B. fusca* larvae; the immature wasps hatch and proceed to consume the host tissues (Polaszek and Walker 1991). Notably, *B. fusca* are not susceptible to infection by strains of *C. sesamiae* from the coast because larvae can mount a successful immune response using encapsulation (Mochiah et al. 2002). The roles of genes involved in mediating immune response to *C. sesamiae* could be investigated in future work.

The *B. fusca* genome harbors a large amount of repetitive sequence (49.8% or 245.5 Mb)—the second highest amount among Lepidopteran genomes sequenced to date (supplementary table S3, Supplementary Material online). In fact, repeat content of *B. fusca* is higher than would be expected based on its genome size (see Talla et al. 2017). LTRs comprise the majority of the repetitive content, occupying 155 Mb (or 31.5%) of the genome (supplementary table S4, Supplementary Material online). This predominance of LTRs is not common among Lepidopterans, though a similar pattern is observed in *P. xylostella*, where the LTR expansion is associated with duplication of genes related to metabolic detoxification (You et al. 2013).

## Conclusion

Our draft genome sequence provides a foundation for future genetic and genomic research on an important crop pest species. We identified a number of loci of interest for pest management, including genes potentially involved in egg structure, chemoreception, dispersal, and immunity. Future work may utilize these insights to develop control measures that can be deployed as part of an integrated pest management strategy to reduce *B. fusca*’s impact on food security.

## Supplementary Material

Supplementary data are available at *Genome Biology and Evolution* online. 

## Supplementary Material

evz166_Supplementary_Data

## Notes

**Data deposition:** This project has been deposited at GenBank under the accession PRJNA553865.
