# Supplementary material for: Draft Genome of Busseola fusca, the Maize Stalk Borer, a Major Crop Pest in Sub-Saharan Africa
Source: Genome Biol Evol. 2019 Jul 31;11(8):2203–7. doi: 10.1093/gbe/evz166 (PMC6697066; doi:10.1093/gbe/evz166)
Supplement: evz166_Supplementary_Data [file evz166_supplementary_data.zip › suppl materials legends.docx]

**Supplemental Materials**

**Figure S1.** Photo of an adult *Busseola fusca*.

**Figure S2.** GO term categories for annotated genes in *Busseola fusca*.

**Figure S3.** Proteome sequence similarity across select species in the order Lepidoptera based on using OrthoMCL to identify clusters of orthologous sequences among *B. fusca*, *B. mori*, *M. sexta*, and *P. xylostella*. The venn diagram shows the numbers of proteins contained within overlapping sequence clusters among species.

**Table S1.** Sample information for *Busseola fusca* DNA sequencing libraries.

**Table S2.** Assembly statistics (number of contigs, N50, and longest sequence) and percent of conserved single-copy orthologs (complete/fragmented/total) identified using the program BUSCO against three databases (Eukaryota, Arthropoda, and Insecta) from the genome assemblies of *Busseola fusca*, *Bombyx mori*, *Manduca sexta*, and *Plutella xylostella*.

**Table S3.** Genome size, gene number, and repeat content for select sequenced Lepidopteran genomes and *Busseola fusca.*

**Table S4.** Detailed information on repeat content in the *Busseola fusca* genome.

**Data File S1.** MAKER-generated gff with gene annotations for *Busseola fusca*.

**Data File S2.** MAKER-generated protein sequences for *Busseola fusca.*

**Data File S3.** List of genes in putatively expanded gene families in *Busseola fusca*.

**Data File S4.** List of genes belonging to gene families that appear to be unique to *Busseola fusca.*

**Data File S5.** Library of repeats generated from the genome of *Busseola fusca.*
